# Biodiesel Production Using K–Sr/CaO and CaO Catalysts Derived from Eggshells by Canola Oil Transesterification

**DOI:** 10.1021/acsomega.4c09118

**Published:** 2025-02-16

**Authors:** Jesús Andrés Tavizón-Pozos, Humberto Cervantes-Cuevas, Germán Gustavo Garcia-Camacho, Gerardo Chavez-Esquivel, Dwight Roberto Acosta-Najarro

**Affiliations:** † Investigadores por México SECIHTI-Área Académica de Química, Departamento de Ciencias Básicas, Universidad Autónoma Metropolitana Azcapotzalco, Avenue San Pablo No. 420, Nueva el Rosario, Azcapotzalco, Ciudad de México 02128, México; ‡ Área Académica de Química, Departamento de Ciencias Básicas, Universidad Autónoma Metropolitana Azcapotzalco, Avenue San Pablo No. 420, Nueva el Rosario, Azcapotzalco, Ciudad de México 02128, México; § Instituto de Física, Circuito de la Investigación Científica, Universidad Nacional Autónoma de México, Ciudad Universitaria, Coyoacán, Ciudad de México 04510, México

## Abstract

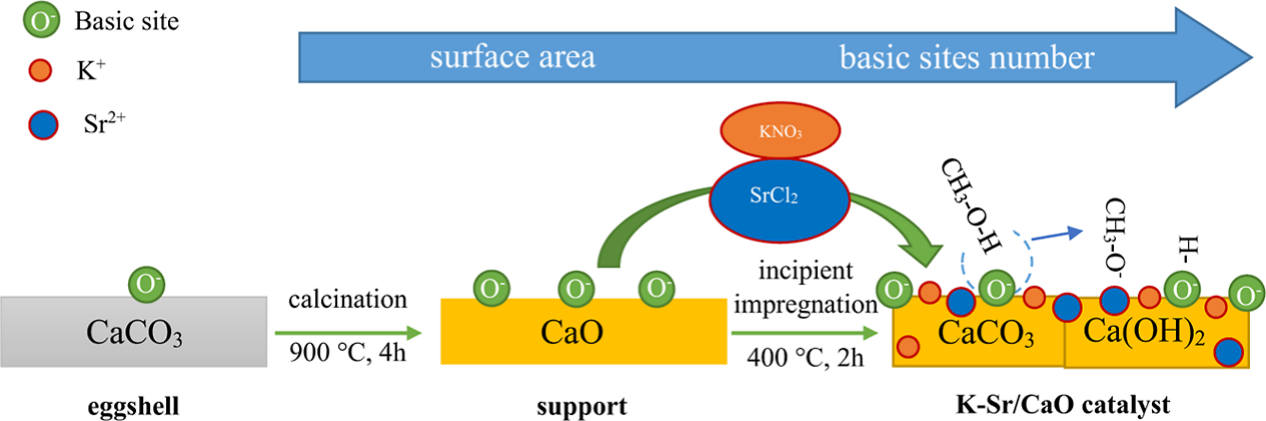

Eggshell calcination at 900 °C was used to produce CaO, which was afterward impregnated with K and Sr using KNO_3_ and SrCl_2_·6H_2_O precursors, diluted in methanol, to improve basicity, stability, and catalytic activity. The CaO doping with K–Sr affected the final catalyst’s textural properties, alkalinity, and basic strength due to the K^+^ and Sr^2+^ size and incorporation into the CaO lattice. SEM images with elemental mapping showed a uniform K^+^ and Sr^2+^ distribution for the K–Sr/CaO catalyst. However, carbonization modified the basic strength and the number of catalytic sites. The fresh K–Sr/CaO and CaO catalysts presented 92.5% and 46% biodiesel yields, respectively. In the third reaction cycle, the biodiesel yield dropped to approximately 72% and 21%, respectively. In this sense, the method of doping CaO with K and Sr increased the basic strength and number of basic sites for the K–Sr/CaO catalyst, providing higher resistance to leaching compared to the CaO catalyst. Finally, the enhanced conditions were 7.0 wt % catalyst loading, a 12.5:1 methanol/oil molar ratio, 70 °C, and a 1 h reaction time.

## 1 Introduction

Biodiesel from vegetable oils has been studied in recent years since it is a fast and economical way to produce biofuels. Nonetheless, there are still challenges to overcome as catalyst performance and recovery need improvement to decrease the environmental impact since conventional catalysts like NaOH are corrosive and difficult to reuse. In this sense, basic or acidic heterogeneous catalysts have emerged as a solution since they provide the sites to facilitate the transesterification of triglycerides or the esterification of free fatty acids, respectively. Heterogeneous catalysts are noncorrosive, and their separation from the mixture is simple. A wide range of basic, acidic, magnetic, and synthetic amino acid-derived catalysts have been suggested in the literature.^1−3^ Independently of its character, one of the major challenges is that they must have highly strong active sites because their activity is relatively limited by mass transfer from the reactants. In addition, a green chemistry and circular economic approach suggests that catalysts should be low-cost, readily available, and harmless. The most widely used heterogeneous catalysts in transesterification reactions are CaO, MgO, and SrO.^4^ Additionally, heterogeneous catalysts based on hierarchical meso/microporous oxide metals have been employed due to their large surface area, large pore volume, and easy adjustment of active sites.^5^ These materials have strong basic catalytic sites, identified as M^+^ and O^–^ superficial pair species that catalyze the methanolysis reaction during transesterification. The strength of active sites in catalysts depends on the synthesis method and precursors, which are responsible for the catalyst’s morphological properties and crystallinity.^6^ It has been accepted that the superficial mechanism occurs by previous methanolysis by the basic sites taking up the H^+^ from the alcohol. Then, the methoxy group adsorbs onto the metallic site and attacks a nearby triglyceride, which may be free or already adsorbed on another site. The nucleophilic attack of the methoxy on the carbonyl group forms a cationic intermediate that, following arrangement, results in a diglyceride and a fatty acid methyl ester (FAME, biodiesel) molecule. Hence, the reaction repeats twice to generate three FAME molecules and one glycerol molecule. The overall reaction could be written as
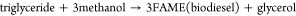


CaO is the most studied material since it exhibits a strong alkaline character. It is thermostable and can be easily obtained from abundant natural sources such as oyster shells, bones, and eggshells.^7,8^ Chicken eggshells are considered waste from restaurants and the food industry, making them an economical CaO source since they mainly contain CaCO_3_ (96%) and trace amounts of Mg, P, and other elements.^9^ Eggshell-based CaO achieved biodiesel yields comparable to those of commercial CaO in rapeseed oil transesterification (95–96%) under the following operational conditions: a 9:1 methanol/oil molar ratio, 1 h reaction time, 4.0 wt % catalyst loading, and 60 °C.^10^ Biodiesel produced by *Argemone mexicana* oil transesterification using an eggshell-CaO catalyst achieved a 99.07% yield using a 9.7:1 methanol/oil molar ratio, 3.05 wt % catalyst loading, and 3 h.^11^ Biodiesel production from waste cooking oil using an eggshell-CaO catalyst achieved a 98.6% yield using a 8:1 methanol/oil molar ratio, 6.0 wt % catalyst loading, 39 min, 55 °C, and 299.7 W power.^12^ The maximum yield of fatty acid methyl esters was 93.5% using an eggshell-CaO catalyst with a 12:1 methanol/oil molar ratio, 5.0 wt % catalyst loading, 65 °C, and 1.5 h.^13^ A biodiesel yield of 96.0% was obtained from waste scum oil containing triglycerides of fatty acids using an eggshell-CaO catalyst with a 6:1 methanol/oil molar ratio, 2.4 wt % catalyst loading, and 65 °C for 3 h under operating conditions. The reusability of the catalyst was also studied, showing that in one, two, three, and four cycles, the biodiesel yield decreased from 96% to 76%, 53%, and 24%, respectively.^14^ In all these cases, the operating conditions, including the methanol/oil molar ratio, reaction temperature, reaction time, and catalyst loading, are determined by the catalytic system.^15,16^ To enhance the stability and catalytic capacity of CaO, doping it with alkali and alkaline earth metals has been suggested.^17^ The K/CaO catalytic system has been tested in palm oil transesterification.^18^ The presence of potassium increased the basicity, leading to an 87.9% biodiesel yield at 80 °C with a 15:1 methanol/oil molar ratio for 2 h. Additionally, this study showed that catalysts deactivate not only due to the leaching of the active sites but also because of glyceroxide formation. In this sense, catalysts could be calcined at 800 °C to restore the basic active sites. On the other hand, SrO is an adequate material for transesterification since it presents a stronger alkali character than CaO. Furthermore, the combination of SrO and CaO has improved the biodiesel yield compared to CaO.^19,20^ Hence, the combination of Sr and K (using KNO_3_, KHCO_3,_ and Sr(NO_3_)_2_ as precursors) on CaO was proposed as promoters for quantity and basic strength.^21^ It was found that the K precursor salt used resulted in a high alkaline character, and a K_2_Sr(CO_3_)_2_ phase was formed. Moreover, it was demonstrated that this carbonate phase was active, and the proportions of Sr and K play a significant role in determining the crystal size of the active phase. Using a Box-Behnken surface response method, the SrK/CaO oyster shell-derived catalysts demonstrated that biodiesel yields above 80% can be achieved using canola oil, with 8.0 wt % catalyst loading, a methanol-to-oil molar ratio of 15, 46 °C, and 3 h. Furthermore, the SrK/CaO oyster shell-based catalysts were active when used with wasted cooking oil but appeared to be easily leachable.^22^ Therefore, the catalytic system K–Sr has demonstrated the potential for use in transesterification reactions. However, stability is still an issue that needs to be addressed. In this sense, this work proposes using KNO_3_ and SrCl_2_·6H_2_O as precursor salts to modify CaO (eggshell-based) to improve the basic strength of the number of active sites while reducing catalyst leaching. Additionally, textural and structural properties were determined to have a better understanding of this catalytic system.

## 2 Materials and Methods

### 2.1 Chemicals and Materials

Strontium chloride hexahydrate (SrCl_2_·6H_2_O, 99% Merck Sigma-Aldrich) and potassium nitrate (KNO_3_, 98% CTR Scientific), methanol (99%, JT Baker), benzene (99%, Merck Sigma-Aldrich), and benzoic acid (99%, Meyer). Chicken eggshell and canola oil were acquired at a local market in México. Hammett indicators: phenolphthalein (p*K*_a_ = 9.1), alizarine yellow (p*K*_a_ = 10.7), benzene (99%, Merck Sigma-Aldrich), benzoic acid (99%, Meyer).

### 2.2 Catalyst Synthesis

The eggshell waste was cleaned with abundant water and 20 mL/g_eggshell_ acetic acid at 65 °C for 1 h to remove organic matter. Then, the eggshells were dried at 80 °C for 48 h to ease the grounding into fine powders and calcined at 900 °C for 5 h. The K–Sr/CaO catalyst was impregnated with 2.5 wt % K and 2.5 wt % Sr using KNO_3_ and SrCl_2_·6H_2_O as precursor salts. The CaO and the adequate amount of each salt were mixed in 15 mL/g_eggshell_ of methanol at room temperature with constant agitation for 1 h. Then, the catalysts were dried at 60 °C overnight and calcined at 400 °C for 3 h.^15,23^

### 2.3 Catalyst Characterization

The surface area (BET), volume, and pore diameter of the CaO and K–Sr/CaO catalysts were obtained by nitrogen physisorption using Autosorb I equipment (Quantachrome). Previously, both catalysts were degassed at 400 °C at a 0.79 Pa vacuum pressure. The FTIR spectra were obtained with Bruker equipment, Lufkin, TX, and added to an ATR accessory. The CaO and K–Sr/CaO catalysts were analyzed using a Siemens D-500 diffractometer with Cu Kα radiation (λ = 0.15406 nm) over a 2θ = 15–70° range. The scan was conducted at 0.001° s^–1^ scan speed, under operating conditions of 35 kV and 20 mV, with a nickel filter employed to enhance measurement accuracy. For X-ray photoelectron spectroscopy (XPS), a PHI 5000 VersaProbe II microprobe was used with an Al Kα X-ray source (*h*ν = 1483.6 eV). The surface of the samples was etched for 2 min at 3.0 kV with Ar^+^ at 0.06 μAmm^–2^. The positions of the peaks were referenced to the high-resolution C 1s region at 284.50 eV as the central peak. Finally, the morphological and compositional analyses of the CaO and K–Sr/CaO catalysts were determined by Scanning Electron Microscopy (SEM) using JEOL 7800F equipment, with 15 kV secondary electronic radiation (LED), 2500×, 5000×, and 10,000× magnifications, and a 12.7 mm working distance.

### 2.4 Catalytic Evaluation

The reaction system for producing biodiesel consists of a batch reactor consisting of a flat-bottom ball flask of 50 mL placed in a heat jacket with magnetic agitation coupled to a water refrigerant-reflux system, which consisted of a condenser as is shown in Figure S1. First, the catalysts were heated to 120 °C for 1 h before the reaction. Then, the catalyst was mixed with methanol to activate it at reaction temperature. After 10 min, the oil was added to the mixture to start the reaction. The reaction parameters were investigated, such as temperature (50, 60, and 70 °C), methanol/oil molar ratio (10:1, 12.5:1, and 15:1), catalyst loading (3, 5, 7, and 11 wt %), and reaction time (0.5, 1.0, and 1.5 h). The temperature effect was carried out with a 7.0 wt % catalyst loading, a 12:1 methanol/oil molar ratio, and a 1 h reaction time, employing the K–Sr/CaO catalyst. The effect of time was at 70 °C, a 12:1 methanol/oil molar ratio, 7.0 wt % catalyst loaded, and 1 h. Each reaction was carried out in independent batches. After the reaction, the mixture was centrifuged at 3000 rpm for 10 min to separate the solid and liquid phases. These latter were poured into a separating funnel, and 2 mL of *n*-hexane was added to accelerate the separation process. After separation, the biodiesel was heated to 60 °C to evaporate the remaining methanol and *n*-hexane. The biodiesel was then washed with to eliminate the lixiviated catalysts that remained in it. The biodiesel yield was measured by a refractive index based on a calibration curve.^24^ The biodiesel density, viscosity, and acid number were measured as is indicated in the Supporting Information. The recovered catalyst was washed twice with 10 mL of *n*-hexane with constant agitation to separate the nonpolar residues. Subsequently, the catalyst was washed twice with 10 mL of methanol to remove polar residues. It was then dried at 120 °C for 24 h and reused in a subsequent cycle.

## 3 Results and Discussion

### 3.1 N_2_ Physisorption

**Figure 1:**
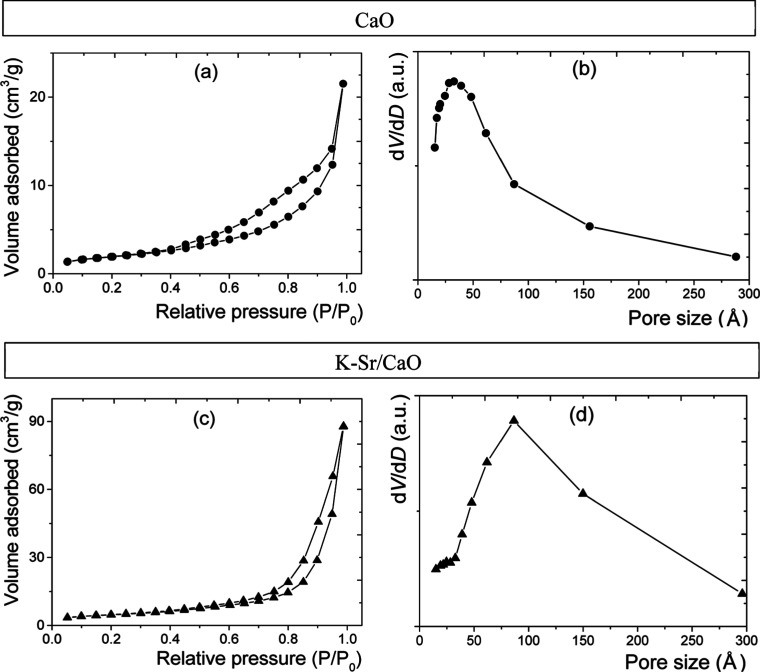
Nitrogen physisorption isotherms and pore size distribution curves of the (*a*–*b*) CaO and (*c*–*d*) K–Sr/CaO catalysts, respectively.

Figure 1 shows the nitrogen physisorption isotherms and pore size distribution curves of the (*a*–*b*) CaO and (*c*–*d*) K–Sr/CaO catalysts, respectively. The observed hysteresis loops in both catalysts corresponded to type IVa N_2_ adsorption isotherm close to type H3 and type H4. This indicated the presence of mesopores, corresponding to irregularly shaped pores with a narrow slit-shaped.

Table 1 shows the textural properties of CaO and K–Sr/CaO catalysts. It was observed that K–Sr doping of CaO increased the surface area, pore volume, and pore diameter by 2.3-fold, 4-fold, and 2.7-fold, respectively. The microtextural properties of similar CaO-based materials were changed by modifying the calcination temperature to change the bimodal pore size distributions (micropores and mesopores) and porosity, specific surface area, and pore radius.^25^ In this sense, not only the precursors during CaO doping can modify the textural properties of the material but also the calcination temperature. For CaO and K–Sr/CaO catalysts, the textural properties obtained were within the domain of mesoporous materials, i.e., suitable for transesterification reactions.

**Table 1:** Surface Area (m^2^/g), Total Pore Volume (cm^3^/g), and Pore Diameter (nm) of the CaO and K–Sr/CaO Catalysts

catalysts	BET surface area ±10% (m^2^/g)	pore volume ±5% (cm^3^/g)	pore diameter ±3% (nm)
CaO	7.0	0.035	17
K–Sr/CaO	16.5	0.141	46

### 3.2 FTIR Spectroscopy

**Figure 2:**
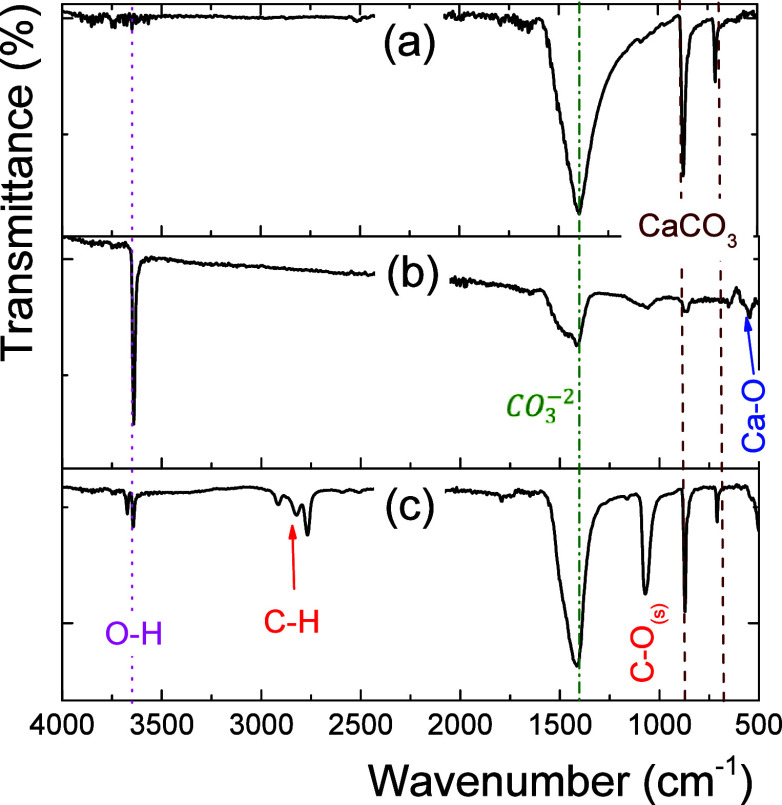
FTIR spectra of the (a) eggshell without calcination, (b) CaO and (c) K–Sr/CaO catalysts.

The FTIR spectra of the Figure 2a chicken eggshell, Figure 2b CaO, and Figure 2c K–Sr/CaO are shown. The uncalcined eggshell showed C–O vibration bands at 1400 (ν_3_) asymmetric out-of-plane, 875(ν_2_) asymmetric in-plane, and 713 (ν_4_) cm^–1^ bending.^26^ At 3640 cm^–1^, the adsorption band agrees with the O–H_(s)_ vibration of Ca(OH)_2_. CaO showed the vibration modes of C–O_(as)_ out-of-plane bending at 1412 and 875 cm^–1^, plus a band at 550 cm^–1^ corresponding to Ca–O vibration.^27^ Finally, the FTIR spectrum of the K–Sr/CaO catalyst shows vibration bands similar to those on CaO. Furthermore, between 2850 and 2700 cm^–1^, three low transmittance bands associated with the C–H vibration mode were observed. These signals could be due to calcium methoxide and organic impurities from eggshells after calcination. At 1080 cm^–1^, it shows a medium band attributed to symmetric C–O stretching vibration, indicating more carbonate groups.

### 3.3 X-ray Diffraction (XRD)

**Figure 3:**
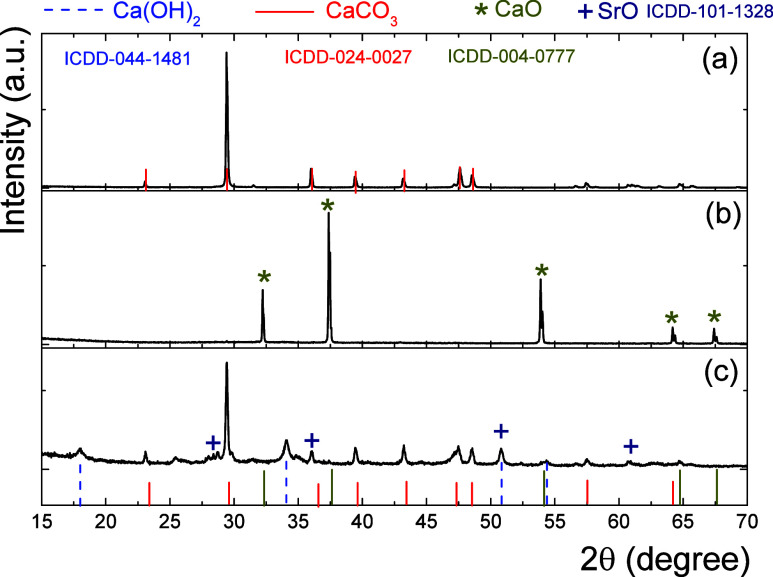
X-ray diffraction spectra of the (a) eggshell without calcination, (b) CaO and (c) K–Sr/CaO catalysts.

The XRD patterns of Figure 3a eggshells, Figure 3b CaO, and Figure 3c K–Sr/CaO catalysts are shown. The eggshell diffractogram showed six diffraction peaks at 22.9° (012), 28.7° (104), 35.0° (110), 39.9° (113), 47.3° (024), and 48.0° (018) associated with CaCO_3_ crystals and verified with the ICDD-024-0027 card for calcite. At 900 °C of calcination, the eggshell residues transitioned from CaCO_3_ and Ca(OH)_2_, into CaO.^28^ In this sense, the CaO catalysts presented diffraction signals of the cubic lime phase at 32° (111), 37.35° (200), 53.86° (220), 64.2° (311), and 66.9° (222) verified with the ICDD-004-0777 card.^18^

The diffractogram of the K–Sr/CaO catalyst showed three crystallographic phases: calcite (CaCO_3_), portlandite (Ca(OH)_2_), and halite cubic (SrO). The diffraction peaks at 17.9, 34.0, 50.7, and 54.3° are associated with portlandite verified with the ICDD-044-1481. After calcination, the hygroscopic character of the materials generated Ca(OH)_2_ crystals.^29^ Also, 28.3° (110), 36.2° (200), 50.7° (220), and 60.8° (311) are associated with cubic SrO verified with the ICDD-101–1328. The coexistence of crystallographic phases in the K–Sr/CaO catalyst can influence the basicity and the strength of the active catalytic sites. Using methanol could generate methoxides during the impregnation of CaO with the precursors of K and Sr. Therefore, during calcination, the methoxides caused new carbonate species, preventing the observation of the rock-salt crystallographic phase of CaO. In general, catalysts with crystallographic phase mixtures can generate high interconnectivity between the networks and planes of each crystalline system, generating surface defects and possible active catalytic sites. The recovered noncalcined Sr–K/CaO catalyst XRD profile is exposed in Figure S2 Supporting Information. The profile showed that highly crystallized CaCO_3_ and calcium methoxide (Ca(OCH_3_)_2_) were observed after one cycle. The Ca(OH)_2_ and CaO crystals were not visible, indicating that these compounds would transform into Ca(OCH_3_)_2_ during the reaction. Based on this, Figure S3 illustrates the possibly predominant crystalline phases of the K–Sr/CaO catalyst, highlighting the not presence of K and Sr ions on the CaO surface. This does not mean that the catalyst has leached, but that the crystals are small and well dispersed on the surface.

### 3.4 Total Basic and Strength Sites

Table 2 shows the total number of basic sites and basic strength for the CaO and K–Sr/CaO catalysts calculated by Hammett indicators.^30^ The basicity results showed that the K–Sr/CaO catalyst presented 2.3 times a higher number of basic sites than the CaO catalyst.

**Table 2:** Total Number of Basic Sites and Basic Strength of the CaO and K–Sr/CaO Catalysts

catalysts	total basic sites (meq_NaOH_/g) ±0.3	basicity
CaO	2.6	6.7 ≤ H_ ≤ 12.1
K–Sr/CaO	5.9	12.1 ≤ H_ ≤ 15.1

Hence, K–Sr incorporation increased the basic site number and strength.^21^ Doping of CaO with Sr and K precursors introduces defects in the calcium oxide crystal lattice, generating potential catalytic sites. Furthermore, alkaline and alkaline earth precursors can modify the basicity of the catalytic sites, which is caused by a greater number of surface oxygen vacancies.^31^ The basic strength increment can be associated with different ionic distances and interactions during Sr^2+^ and K^+^ incorporation into CaO.^32^ A strong basic character can be formed by strontium introduction into the CaO lattice since the ionic radii of Sr^2+^ are smaller than K^+^ (1.18 Å < 1.37 Å). In contrast, potassium particles could be available on the surface of CaO.^33^ The K–Sr/CaO catalyst presents the coexistence of three crystallographic phases obtained by the incipient impregnation-calcination at 400 °C. The percentage abundance of each crystallographic phase, from highest to lowest, had the following sequence: CaCO_3_> Ca(OH)_2_ > SrO. The dominant basic strength of this catalyst is a function of the basic forces of the dominant crystallographic phases. In this sense, the alkaline strength is calculated based on the p*K*_b_ value of CaCO_3_, which is 21% lower than the p*K*_b_ value of Ca(OH)_2_.

### 3.5 X-ray Photoelectron Spectroscopy (XPS)

**Figure 4:**
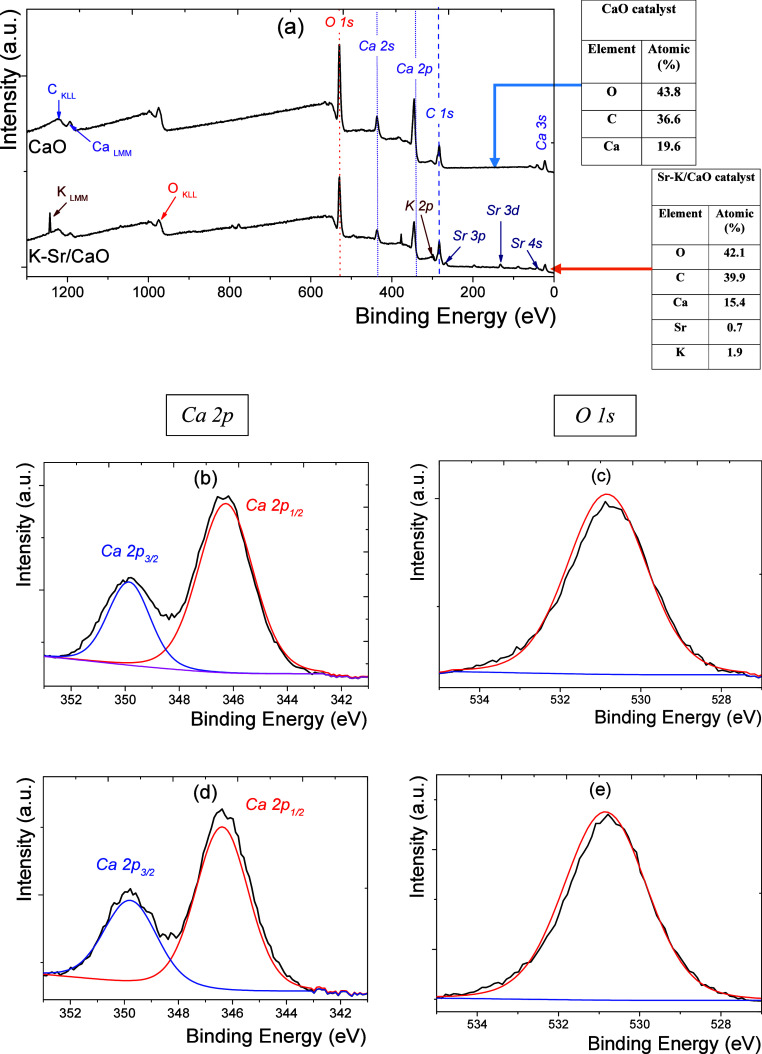
XPS survey spectra for (a) CaO and K–Sr/CaO catalysts. High-resolution deconvoluted XPS spectra for the Ca 1 s (left) and O 1 s (right) regions of the (b,c) CaO and (d,e) K–Sr,/CaO catalysts.

Figure 4a shows the XPS survey spectra of the CaO and K–Sr/CaO catalysts. The analysis revealed that the surface of both catalysts is composed of oxygen, carbon, calcium, potassium, and strontium. The presence of carbon can be attributed to the presence of carbonate in the catalyst surface.^34^ At 346.28 and 349.89 eV, the high-resolution region of Ca 2p corresponds to Ca 2p_1/2_ and Ca 2p_3/2_.^35,36^

The ratio Ca 2p_1/2_ /Ca 2p_3/2_ was 2.2 and 2.0 for CaO (Figure 4b) and K–Sr/CaO (Figure 4d), respectively. The minimal difference is associated with the same oxidation state, the chemical environment, and the nature of the bonds of the calcium atoms in both catalysts. The high-resolution region of O 1s for CaO (Figure 4c) and K–Sr/CaO (Figure 4e) at 530.84 eV corresponds to the oxygen of the metal oxide network.^37^

However, the amount of surface oxygen for the K–Sr/CaO catalyst was 9.8% lower than that obtained for CaO. Low oxygen levels on the catalyst surface can create highly reactive oxygen vacancies. This can also change the adsorption energies of canola oil and the intermediates generated during the transesterification reaction. Also, this band can be assignable to O^2–^ in Ca–O bonds.^38^

### 3.6 Scanning Electron Microscopy (SEM) with Energy Dispersive X-ray Analysis (EDX)

**Figure 5:**
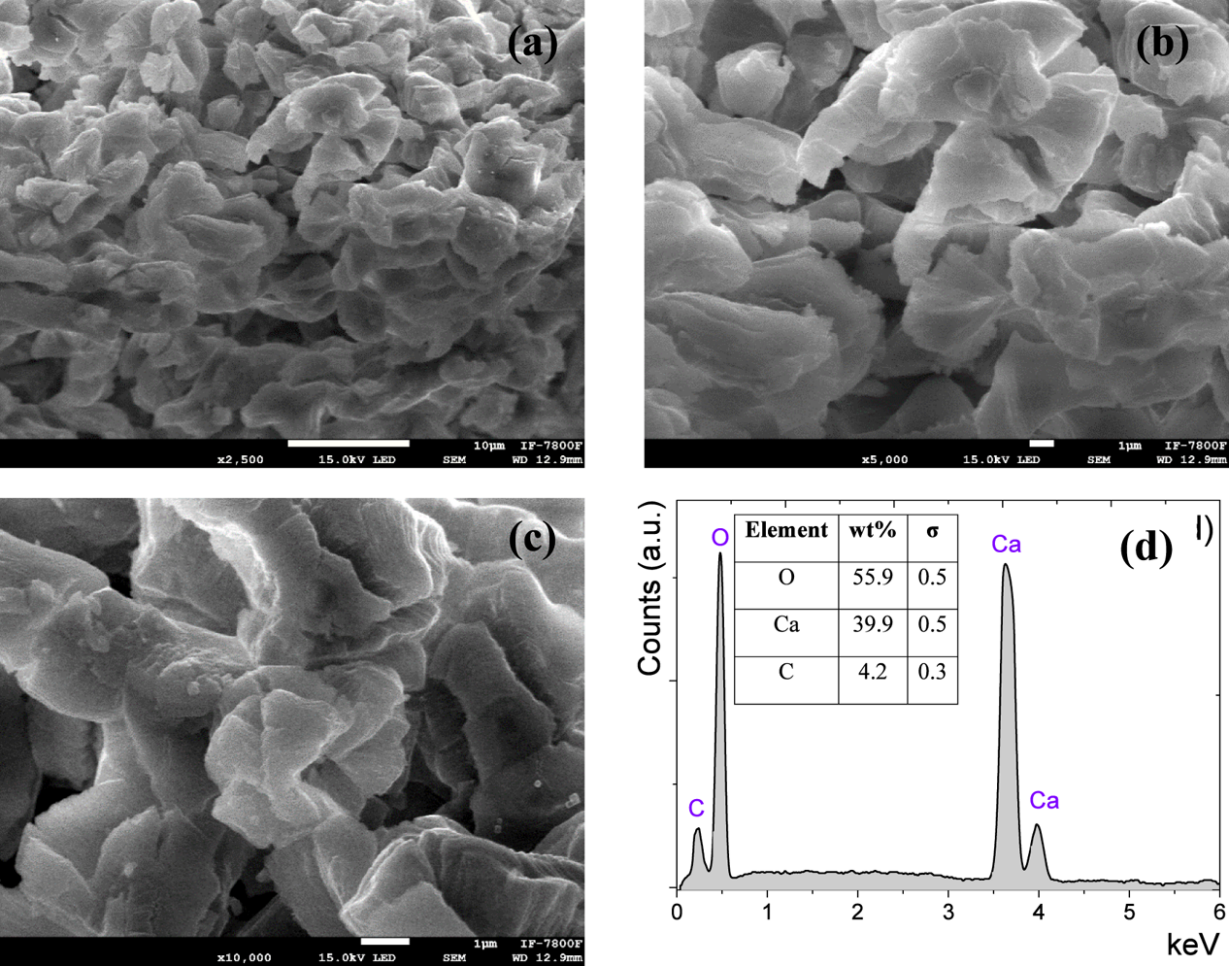
Scanning electron microscopy micrographs at (a) 2500×, (b) 5000×, (c) 10,000× magnifications, and (e) EDX analysis for CaO catalyst.

The SEM micrographs of the CaO catalyst (Figure 5) showed a honeycomb-like structure generating superficial pores with a specific location on the surface of the material; the atomic percentages of Ca and O were 39.9% and 55.9%, respectively.^39^

**Figure 6:**
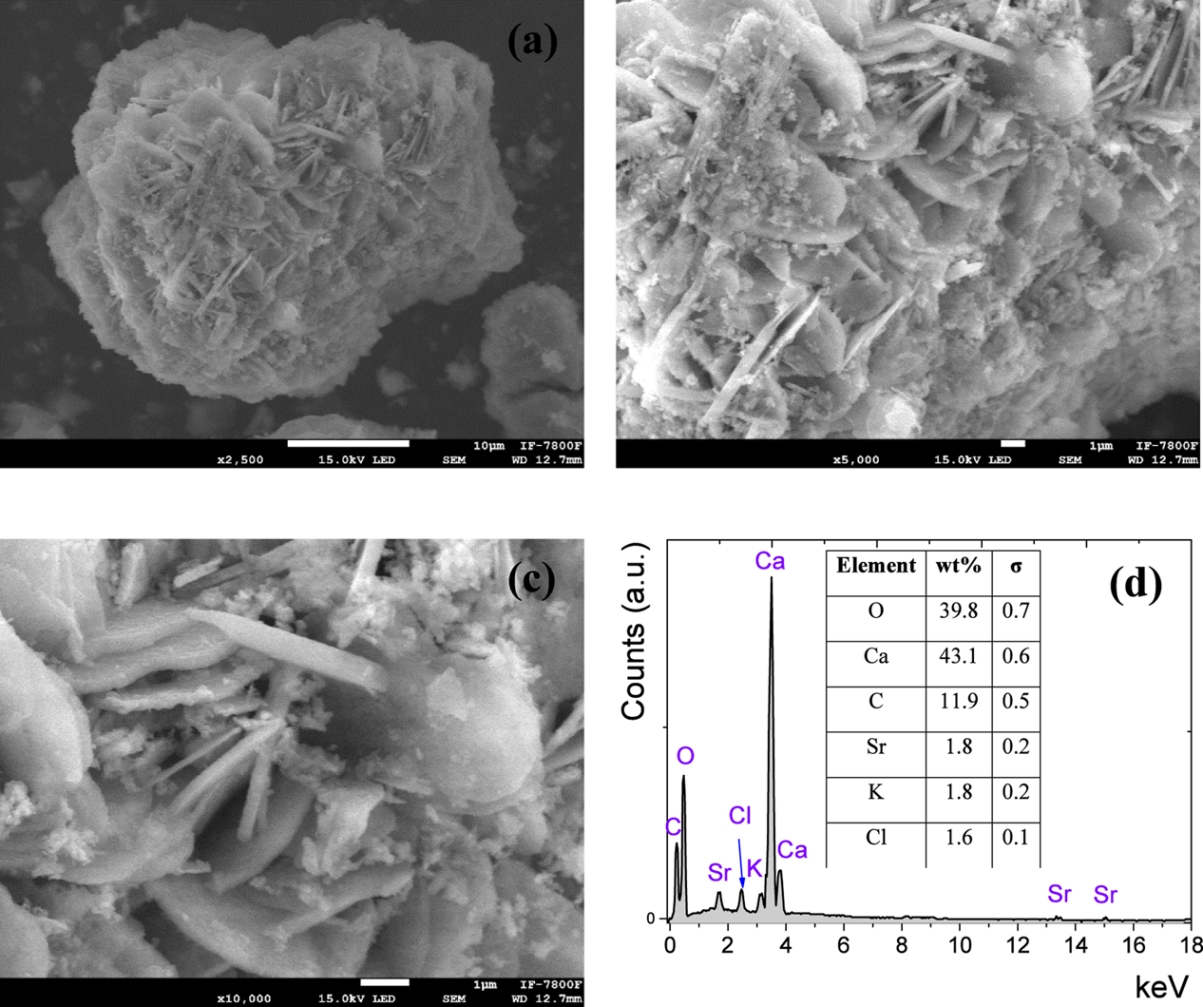
Scanning electron microscopy micrographs at (a) 2500×, (b) 5000×, (c) 10,000× magnifications, and (e) EDX analysis for K–Sr/CaO catalyst.

Figure 6 shows the SEM micrographs and EDX analysis for the K–Sr/CaO catalyst, in which the morphology presented rod-shaped particles with a granulometric distribution of 1.5 and 4.0 μm associated with a mixed Ca(OH)_2_ and CaCO_3_. SEM micrographs for the mixed CaCO_3_–Ca(OH)_2_ system showed rod particles with lengths of ca. 5 μm, where the Ca(OH)_2_ acts as a stabilizer for the calcite particles.^40^ According to the EDX reports, the metal/oxygen ratio was ca. 0.71 and 1.18 for CaO and K–Sr/CaO catalysts, respectively. High metal/oxygen ratios may indicate the presence of active sites that facilitate the adsorption process between oil and methanol.

This indicates that Sr and K doping can affect the CaO crystal structure, producing oxygen defects and surface vacancies. In the K–Sr/CaO catalyst, both metals presented 1.8 wt % on the surface (2.5 wt % theoretical loading). In this sense, 28% of K and Sr would be incorporated into the CaO lattice, leading to the effects observed in the characterization techniques.

**Figure 7:**
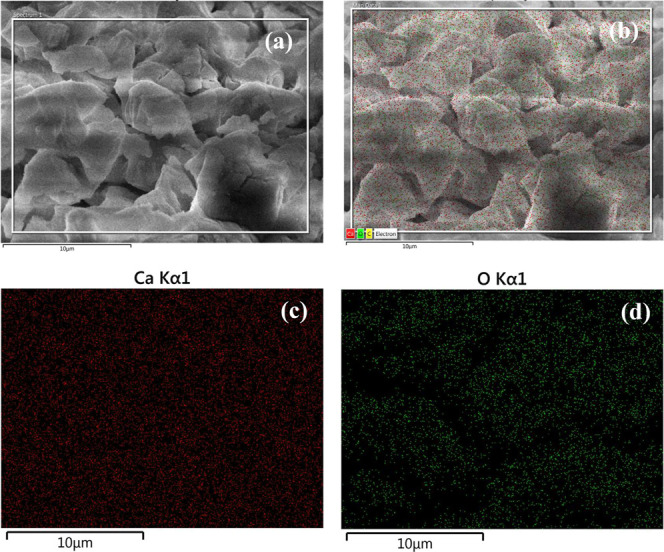
Scanning electron microscopy micrographs with chemical mapping for (a,b) CaO catalyst with (c) red stands for Ca Kα1, and (d) green stands for O Kα1 with 10 μm scale marks.

**Figure 8:**
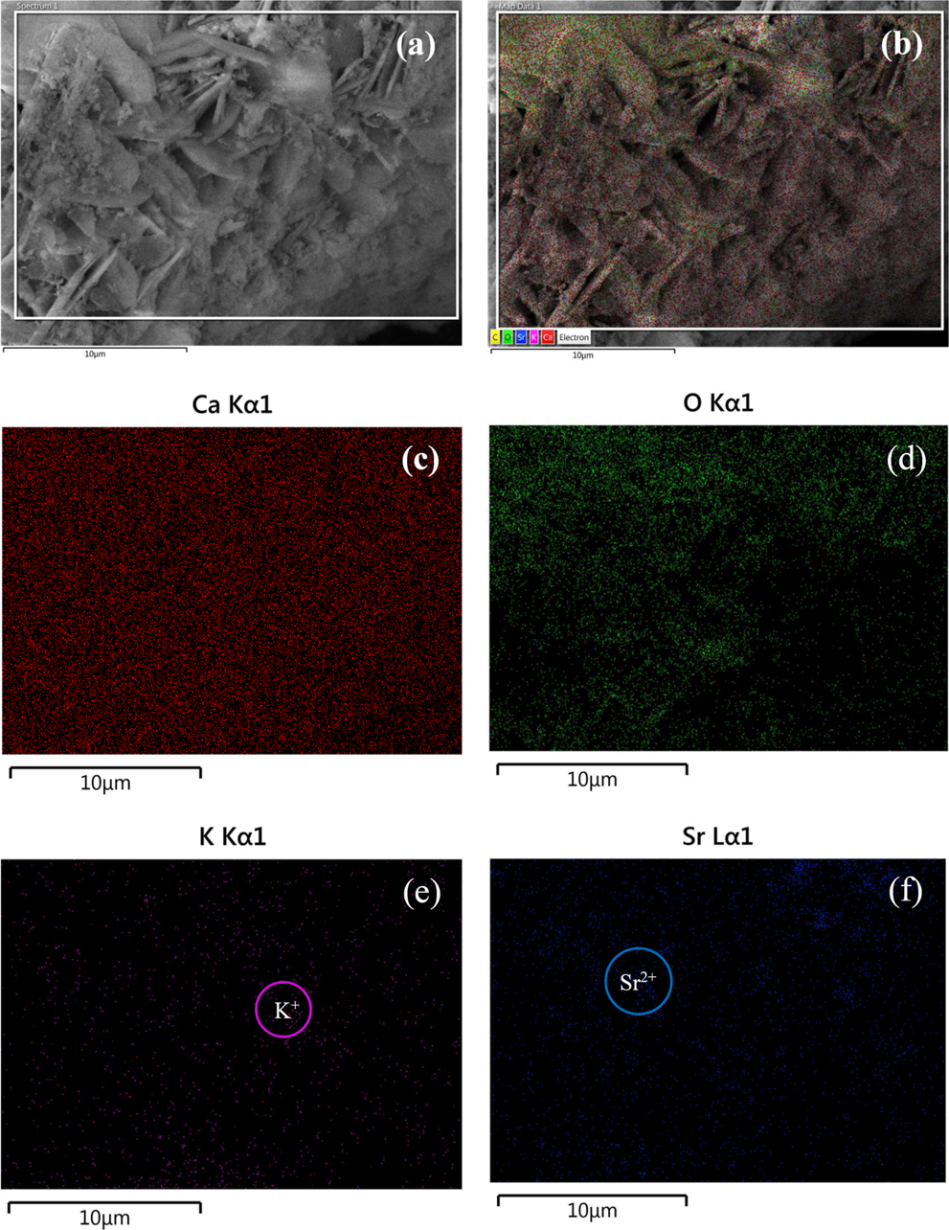
Scanning electron microscopy micrographs with chemical mapping for (a,b) K–Sr/CaO catalyst with (c) red stands for Ca Kα1, (d) green stands for O Kα1, (e) pink stands for K Kα1 and (f) blue stands for Sr Lα1 with 10 μm scale marks.

The SEM images and elemental mapping (chemical composition analysis) for the CaO and K–Sr/CaO catalysts are presented in Figures 7 and 8, respectively. Both micrographs show a homogeneous Ca^2+^, K^+^, and Sr^2+^ distribution on both catalyst surfaces. CaO doped with K–Sr favored high surface areas with the formation and dispersion of active catalytic sites responsible for improved catalytic activity, avoiding cations from aggregating on the surface of the catalyst.^41^

### 3.7 Transesterification Reactions

**Figure 9:**
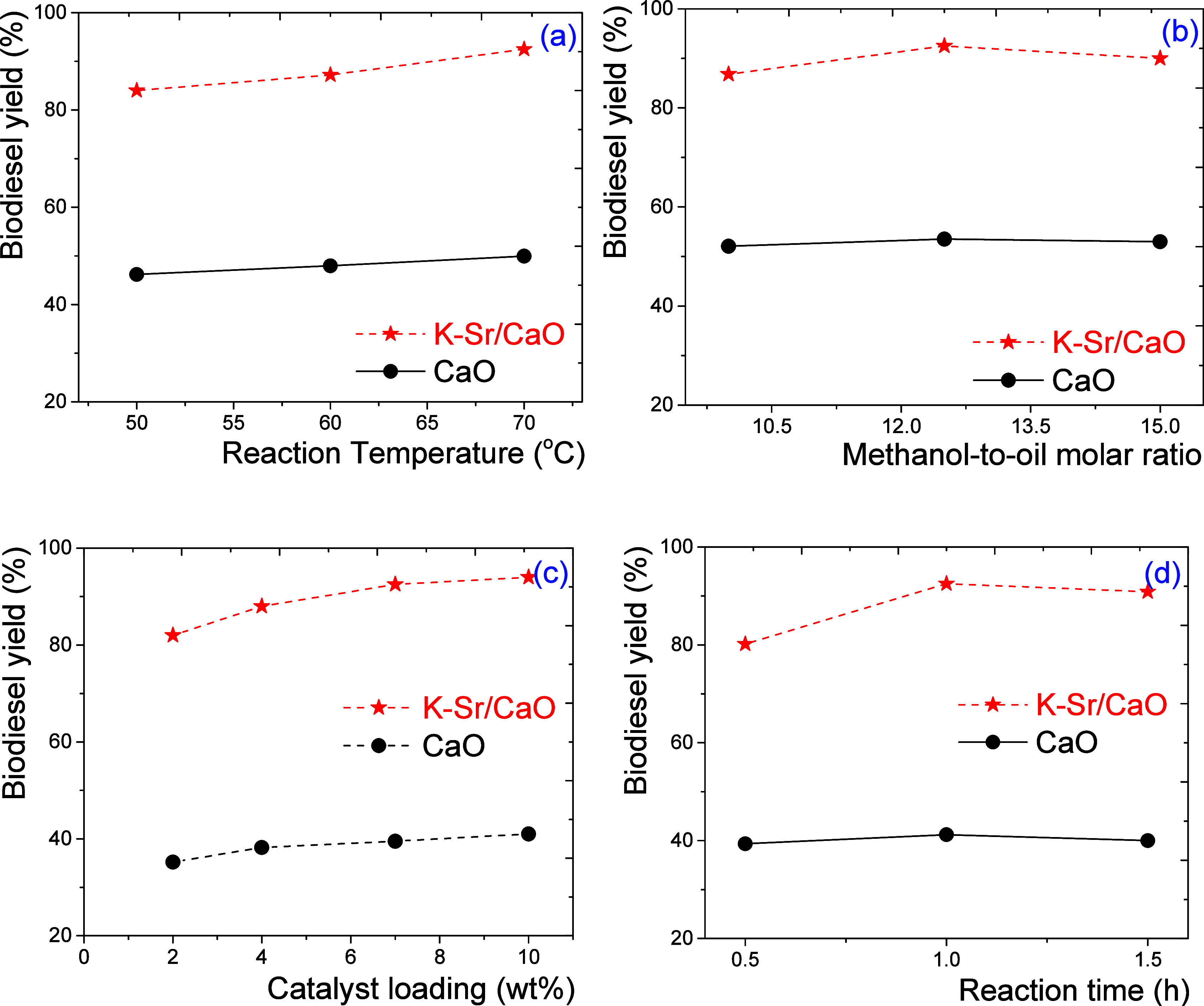
Effect of (a) reaction temperature, (b) methanol/oil molar ratio, (c) catalyst loading, and (d) reaction time as a function of biodiesel yield (%). Reaction conditions: 70 °C (b–d), a 12.5:1 methanol/oil molar ratio (a,c,d), 7.0 wt % catalyst loading (a,b,d), and 1 h of reaction (a–c) using CaO and K–Sr/CaO catalyst in all cases.

The temperature effect in the canola oil transesterification using CaO and K–Sr/CaO catalysts was a 12.5:1 methanol/oil molar ratio, 7.0 wt % catalyst loading, and 1 h reaction time (Figure 9a). The results showed a biodiesel yield of 84% at 50 °C with an increment to 91% at 70 °C using the K–Sr/CaO catalyst, which was 45% higher than those obtained using CaO. The viscosity and density of canola oil decreased at high temperatures, and its operational temperature approached the boiling point of methanol. This facilitates the mobility of the components and adsorption-reaction-desorption on the surface of the catalysts.^42,43^ In this sense, at 70 °C and 1 h of reaction, methanol could mix and react properly with the triglycerides. Hence, a high biodiesel yield was obtained. Figure 9b shows the methanol/oil molar ratio effect in the transesterification reactions. This parameter was evaluated and performed with a constant 7.0 wt % catalyst loading (CaO and K–Sr/CaO) at 70 °C and a 1 h reaction time. The 12.5:1 methanol/oil molar ratio produced a 91% and 53% biodiesel yield using K–Sr/CaO and CaO catalysts, respectively. At higher methanol concentrations, the total volume of the reaction system increases, and the catalyst is more dispersed, decreasing the probability of reaching the active sites. In this sense, a 12.5:1 methanol/oil molar ratio seems adequate for CaO and K–Sr/CaO systems. The use of excessive alcohol promotes glycerol dilution, limiting the methanolysis of the oil. However, since methanol is always in excess, the reaction should be preferred to products.^44^ Hence, more evidence is needed to elucidate the behavior of these reaction systems.

Figure 9c shows the effect of catalyst loading was performed at 70 °C, a 12.5:1 methanol/oil molar ratio, and a 1 h reaction time. The biodiesel yield increased from 82% at 3.0 wt % to 93.5% at 11.0 wt % catalyst loading for the K–Sr/CaO catalyst. More biodiesel yield was obtained as more active sites were in the reaction system.^16^ However, according to the trend in both catalysts, the biodiesel produced above 7.0 wt % catalyst loading seems to remain constant. Finally, the reaction time effect was performed at 70 °C with a 12.5:1 methanol/oil molar ratio and 7.0 wt % catalyst loading. Figure 9d showed a local maximum in 1 h of reaction; the biodiesel yield was 90% and 41% for K–Sr/CaO and CaO catalysts, respectively. Since the boiling point of methanol does not allow the temperature to rise further, there are 10 min during which it is kept at this temperature. After this time, the methanol, biodiesel, and glycerol formed can be mixed, changing the density and viscosity of the mixture. They also allow the temperature to be raised to 70 °C as the reaction progresses. However, at 0.5 h, the time is too short, and at 1.5 h, yield is no significant change. Therefore, 1 h of reaction is sufficient to have high reaction yields. The homogeneous and heterogeneous contributions at these conditions of the Sr–K/CaO catalyst were carried out by mixing it with methanol for 1 h and then filtrated. The methanol with a leached catalyst was set into the reaction with the oil, and the biodiesel yield was 9.87%. This result confirms the lixiviation of the active sites and that those leached species contribute. Nonetheless, it is low compared to the heterogeneous phase. This is evidence of the high activity of the alkaline sites present on the surface. Table 3 compares this catalytic system with Sr/CaO and K/CaO systems. In particular, the K–Sr/CaO catalyst showed an acceptable biodiesel yield, which took only 1.5 h to reach a biodiesel yield of 90%. However, the homogeneous catalyst is more active since it does not present limitations in mass transfer. Likewise, it is observed that Na^+^ and K^+^ ions are active in palm and canola oil transesterification reactions.^18,45^

**Table 3:** Comparison of Eggshell-CaO Systems Used as Catalysts in Biodiesel Production, Fresh Catalyst Results^a^

catalytic system	oil type	operation conditions	biodiesel yield (%)	references
KF/CaO nanocatalyst	Chinese tallow seed oil	CL: 3.0 wt %	96.0	(23)
		M/O: 9:1		
		*T*: 65 °C		
		*t*: 2 h		
Sr(NO_3_)_2_/CaO	cottonseed oil (CO) and waste frying oil (FO)	CL = 3.5 wt %	97.3 (CO)	(46)
		M/O = 12:1	96.7 (FO)	
		*T* = 75 °C		
		*t* = 2 h		
K_2_CO_3_/CaO (nanoparticles)	canola oil	CL: 7.0 wt %	97.7	(47)
		M/O: 9:1		
		*T*: 65 °C		
		*t*: 8 h		
KOH/CaO	soybean oil	CL: 5.0 wt %	98.9	(48)
		M/O: 6:1		
		*T*: 200 °C (800 W)		
		*t* = 15 min		
K_2_O/CaO	palm oil	CL = 3.0 wt %	87.5	(18)
		M/O = 15:1		
		*T* = 80 °C		
		*t* = 2 h		
activated carbon and KOH/CaO	palm oil	CL = 3.0 wt %	85.7	(49)
		M/O = 10:1		
		*T* = 65 °C		
		*t* = 3 h		
K^+^/CaO	waste frying Oil	CL = 3.0 wt %	98.5	(33)
		M/O = 12:1		
		*T* = 65 °C		
		*t* = 3 h		
Na–K/CaO	canola oil	CL: 3.0 wt %	97.6	(50)
		M/O: 9:1		
		*T*: 50 °C		
		*t*: 3 h		
SrO/CaO nanoparticles	waste palm cooking oil	CL: 5.0 wt %	99.3	(45)
		M/O: 9:1		
		*T*: 80 °C		
		*t*: 3 h		
Ca(OCH_3_)_2_/Al_2_O_3_	waste cooking oil	CL: 9.0 wt %	90.0	(51)
		M/O: 12.5:1		
		*T*: 60 °C		
		*t*: 3 h		
CaO–K_2_O	Jatropha Curcas oil	CL: 3.69 wt %	97.1	(52)
		M/O: 19.48:1		
		*T*: 70 °C		
		*t*: 1.8 h		
Sr–K/CaO	canola oil	CL: 7.0 wt %	92.5	this work
		M/O: 12.5:1		
		*T*: 70 °C		
		*t*: 1 h		

a CL = catalyst loading; M/O = methanol/oil molar ratio; *T* = temperature; *t* = time.

Previous work shows that the catalyst loading could be lower than that proposed in this work. However, it requires more time or higher temperature, particularly wasted cooking oils or those with high fatty acid content. Likewise, previous works on K/CaO or Sr/CaO systems require 2 to 3 h, except for the one that uses microwaves as heating, while the system presented in this work only required one hour. The methanol/oil molar ratio (M/O) plays an important role in biodiesel yield, as higher ratios can help drive the reaction toward complete transesterification. The highest M/O ratio in the table, 19.48:1, was used in the CaO–K_2_O system with Jatropha Curcas oil, achieving a 97.1% yield.^52^ On the other hand, the K^+^/CaO system, which utilized a moderate M/O ratio of 12:1, achieved an impressive yield of 98.5% with waste frying oil.^33^ This suggests that an optimized M/O ratio is crucial for achieving high biodiesel yields, but excessively high ratios do not always guarantee the best results. The Sr–K/CaO catalyst in this study (92.5% yield from canola oil under mild conditions) offers a competitive performance compared to other systems, particularly considering its relatively low reaction temperature (70 °C) and shorter reaction time (1 h).

### 3.8 Reusability of the Catalysts

**Figure 10:**
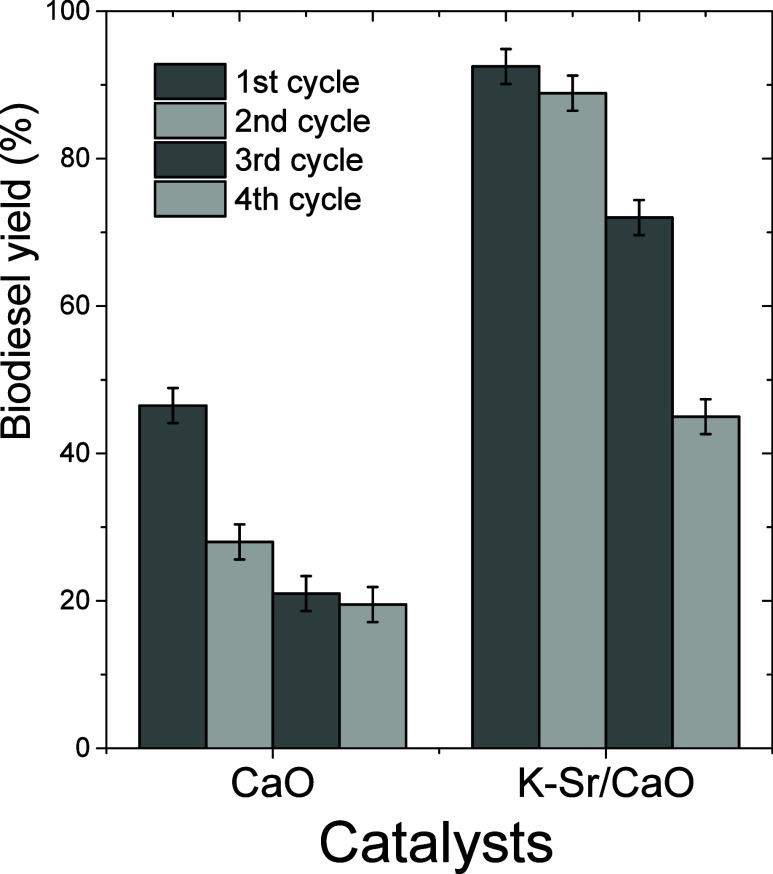
Reusability of CaO and K–Sr/CaO catalysts as a function of biodiesel yield (%) (reaction conditions: 7 wt % catalyst loading, a 12.5:1 methanol/oil molar ratio, 70 °C reaction temperature, and 1 h reaction time).

Figure 10 shows the reusability of both catalysts at 70 °C with a 12.5:1 methanol/oil molar ratio, 7.0 wt % catalyst loading, and a 1 h reaction time. Using a fresh CaO catalyst, the biodiesel yield was 46.5%, decreasing to 19.0% in the fourth reaction cycle. The K–Sr/CaO catalyst remained stable through the third cycle, achieving a biodiesel yield of over 72.0%. After four cycles of canola oil transesterification, both catalysts experienced sequential deactivation, likely due to partial leaching and strong glycerol adsorption on the active sites.^43^ This might be explained by the surface concentrations of Sr and K, as SEM-EDS with chemical mapping results showed. At 400 °C of calcination temperature, SrCl_2_ did not decompose completely, and chloride species are still on the catalyst’s surface.

Hence, its presence may provide not only alkalinity but also stability. This would explain that only 20% of K and Sr are on the surface, and few ions are introduced in the lattice. In this sense, using SrCl_2_ and KNO_3_ calcined at 400 °C provided stability to the catalysts by possibly mitigating the formation of glyceroxides but not avoiding it. Hence, lixiviation and deactivation are still occurring. Nonetheless, the proposed synthesis provided well-defined structures and morphology that limited the lixiviation process in the second cycle. Particularly, the K–Sr/CaO catalyst of this work showed higher stability compared to SrK/CaO synthesized from oyster shells using Sr(NO_3_)_2_ as precursor and calcined at 800 °C.^21^

Table 4 presents the physicochemical properties of biodiesel with an AN 16.5% lower than fresh canola oil. Furthermore, compared to the ASTM D6751 and EN14214 normative criteria, the AN in the synthesized biodiesel dropped by 30%.^53^ All the properties were obtained using a fresh K–Sr/CaO catalyst. The biodiesel-determined values for density and viscosity fall within the quality ranges specified by both normativity requirements.

**Table 4:** Physicochemical Properties of Biodiesel Produced by Canola Oil Transesterification Using a K–Sr/CaO Catalyst

sample	saponification number	acid number	density (15 °C) (g/cm^3^)	viscosity (40 °C) (mm^2^/s)
	(mg_KOH_/g)			
biodiesel	93.4	0.096	0.862	4.94
canola oil	112.75	0.132	0.941	26.57
EN 14214		≤0.5	0.86–0.9	3.5–5
ASTM D6751		≤0.5	0.87–0.89	1.9–6

## 4 Conclusion

CaO and K–Sr/CaO catalysts were synthesized from eggshell wastes, SrCl_2_, and KNO_3_. The doping of CaO with K and Sr salts improved the surface area, pore volume, and number of basic sites, significantly enhancing its catalytic performance in biodiesel production from canola oil. The K–Sr incorporation into CaO causes the calcite, portlandite, and halite phases to coexist. This combination led to a 3-fold increase in surface area and a 2.3-fold increase in basicity compared to CaO, which is associated with more catalytic sites. Furthermore, a significant surface morphological change was observed between CaO and K–Sr/CaO, where doping-induced surface defects are linked to forming potential new catalytic sites. Defects are linked to the production of oxygen vacancies on the surface, resulting in lower oxygen levels for the K–Sr/CaO catalyst than pure CaO. This can be attributed to high reactivity and a remarkable improvement in biodiesel yield, where the fresh K–Sr/CaO catalyst obtained a biodiesel yield of 92.5%, and the second reaction cycle was 89%. The improved catalytic performance of the K–Sr/CaO catalyst can be attributed to the homogeneous Sr^2+^ and K^+^ dispersion on the CaO surface, promoting the strength and stability of the catalytic site. Furthermore, enhanced conditions were found in the transesterification of canola oil using the K–Sr/CaO system with a 7 wt % catalyst loading, a 12.5:1 methanol/oil molar ratio, 70 °C, and a reaction time of 1 h. Overall, the study highlights the potential of using waste materials such as eggshells to develop efficient and stable catalysts for biodiesel production, emphasizing the role of CaO doping with K and Sr in improving catalytic properties. The interactions between the K–Sr precursors and the Ca support play a crucial role in the formation of active species on the catalyst surface. Therefore, it is essential to further understand them by varying factors such as precursor concentration, calcination temperature, and the type of solvent used during the impregnation process. This approach represents a key objective for future studies.

## Supporting Information Available

Contains information on the reaction system, calculations of the saponification number (SN), acid number (AN), diffraction spectrum of the Sr–K/CaO catalyst used, and the schematic representation of the K–Sr/CaO catalyst crystalline phases and methoxyl group formation during the transesterification reaction for biodiesel production (PDF)

## References

[ref1] Hou S.; Xie W. Three-dimensional hierarchical meso/macroporous Mo/Ce/TiO_2_ composites enhances biodiesel production from acidic soybean oil by transesterification-esterifiications. Energy Convers. Manage. 2024, 305, 118273 10.1016/j.enconman.2024.118273.

[ref2] Xie W.; Han Y.; Wang H. Magnetic Fe_3_O_4_/MCM-41 composite-supported sodium silicate as heterogeneous catalysts for biodiesel production. Renewable energy 2018, 125, 675–681. 10.1016/j.renene.2018.03.010.

[ref3] Li J.; Guo Z. Structure evolution of synthetic amino acids-derived basic ionic liquids for catalytic production of biodiesel. ACS Sustainable Chem. Eng. 2017, 5 (1), 1237–1247. 10.1021/acssuschemeng.6b02732.

[ref4] Salamatinia B.; Hashemizadeh I.; Zuhairi A. A. Alkaline Earth Metal Oxide Catalysts for Biodiesel Production from Palm Oil: Elucidation of Process Behaviors and Modeling Using Response Surface Methodology. Iran. J. Chem. Chem. Eng. 2013, 32, 113–126. 10.30492/ijcce.2013.5911.

[ref5] Li K.; Xie W. Enhanced biodiesel production from low-value acidic oils using ordered hierarchical macro–mesoporous MoAl@H-SiO_2_ catalyst. Fuel 2024, 364, 131105 10.1016/j.fuel.2024.131105.

[ref6] Zhang G.; Hattori H.; Tanabe K. Aldol addition of acetone, catalyzed by solid base catalysts: magnesium oxide, calcium oxide, strontium oxide, barium oxide, lanthanum (III) oxide and zirconium oxide. Appl. Catal. 1988, 36, 189–197. 10.1016/S0166-9834(00)80114-1.

[ref7] Moura C. V. R.; Abreu W. C.; Moura E. M.; Costa Jean C. S. Chapter 6—CaO derived from waste shell materials as catalysts in synthesis of biodiesel. In Waste and Biodiesel; Singh B., Guldhe A., Eds.; Elsevier, 2022; pp 91–118. 10.1016/B978-0-12-823958-2.00001-X.

[ref8] Waheed M.; Butt M. S.; Shehzad A.; Adzahan N. M.; Shabbir M. A.; Rasul Suleria H. A.; Aadil R. M. Eggshell calcium: A cheap alternative to expensive supplements. Trends Food Sci. Technol. 2019, 91, 219–230. 10.1016/j.tifs.2019.07.021.

[ref9] Chung Z. L.; Tan Y. H.; Chan Y. S.; Kansedo J.; Mubarak N. M.; Ghasemi M.; Abdullah M. O. Life cycle assessment of waste cooking oil for biodiesel production using waste chicken eggshell derived CaO as catalyst via transesterification. Biocatal. Agric. Biotechnol. 2019, 21, 101317 10.1016/j.bcab.2019.101317.

[ref10] Yaşar F. Biodiesel production via waste eggshell as a low-cost heterogeneous catalyst: Its effects on some critical fuel properties and comparison with CaO. Fuel 2019, 255, 115828 10.1016/j.fuel.2019.115828.

[ref11] Ashine F.; Kiflie Z.; Prabhu S. V.; Tizazu B. Z.; Varadharajan V.; Rajasimman M.; Joo S. W.; Vasseghian Y.; Jayakumar M. Biodiesel production from *Argemone mexicana* oil using chicken eggshell derived CaO catalyst. Fuel 2023, 332 (Part 2), 126166 10.1016/j.fuel.2022.126166.

[ref12] Attari A.; Abbaszadeh-Mayvan A.; Taghizadeh-Alisaraei A. Process optimization of ultrasonic-assisted biodiesel production from waste cooking oil using waste chicken eggshell-derived CaO as a green heterogeneous catalyst. Biomass Bioenergy 2022, 158, 106357 10.1016/j.biombioe.2022.106357.

[ref13] Farooq M.; Ramli A.; Naeem A.; Mahmood T.; Ahmad S.; Humayun M.; Islam M. G. U. Biodiesel production from date seed oil (*Phoenix dactylifera L.*) via egg shell derived heterogeneous catalyst. Chem. Eng. Res. Des. 2018, 132, 644–651. 10.1016/j.cherd.2018.02.002.

[ref14] Kavitha V.; Geetha V.; Jacqueline P. J. Production of biodiesel from dairy waste scum using eggshell waste. Process Saf. Environ. Prot. 2019, 125, 279–287. 10.1016/j.psep.2019.03.021.

[ref15] Odetoye T. E.; Agu J. O.; Ajala E. O. Biodiesel production from poultry wastes: Waste chicken fat and eggshell. J. Environ. Chem. Eng. 2021, 9, 105654 10.1016/j.jece.2021.105654.

[ref16] Erchamo Y. S.; Mamo T. T.; Workneh G. A.; Mekonnen Y. S. Improved biodiesel production from waste cooking oil with mixed methanol-ethanol using enhanced eggshell-derived CaO nanocatalyst. Sci. Rep. 2021, 11, 6708 10.1038/s41598-021-86062-z. 33758293 PMC7988067

[ref17] Olvera D.; Rodriguez J. A.; Perez-Silva I.; Chavez-Esquivel G.; Tavizón-Pozos J. A. Catalytic evaluation of Li and K supported on CaO in the transesterification of triolein, triestearin, and tributyrin. Chem. Pap. 2022, 76, 6287–6295. 10.1007/s11696-022-02305-x.

[ref18] Maneechakr P.; Karnjanakom S. Systematic production of biodiesel fuel from palm oil over porous K_2_O@CaO catalyst derived from waste chicken eggshell via RSM/kinetic/thermodynamic studies. J. Environ. Chem. Eng. 2021, 9, 106542 10.1016/j.jece.2021.106542.

[ref19] Tangy A.; Pulidindi I. N.; Dutta A.; Borenstein A. Strontium oxide nanoparticles for biodiesel production: fundamental insights and recent progress. Energy Fuels 2021, 35, 187–200. 10.1021/acs.energyfuels.0c03815.

[ref20] Tavizón-Pozos J. A.; Cruz-Aburto Z. G. A review of the use of SrO in catalysts for biodiesel production. Biofuels, Bioprod. Biorefin. 2024, 18 (2), 652–668. 10.1002/bbb.2562.

[ref21] Hernández-Martínez M. A.; Rodriguez J. A.; Chavez-Esquivel G.; Ángeles-Beltrán D.; Tavizón-Pozos J. A. Canola oil transesterification for biodiesel production using potassium and strontium supported on calcium oxide catalysts synthesized from oyster shell residues. Next Mater. 2023, 1, 100033 10.1016/j.nxmate.2023.100033.

[ref22] Ramírez-Paredes E. A.; Rodriguez J. A.; Chavez-Esquivel G.; Tavizón-Pozos J. A. Effect of Sr concentration in SrK/CaO oyster shell derived catalysts for biodiesel production. Int. J. Chem. React. Eng. 2024, 22 (6), 689–700. 10.1515/ijcre-2024-0021.

[ref23] Wen L.; Wang Y.; Lu D.; Hu S.; Han H. Preparation of KF/CaO nanocatalyst and its application in biodiesel production from Chinese tallow seed oil. Fuel 2010, 89, 2267–2271. 10.1016/j.fuel.2010.01.028.

[ref24] Li S.; Kwofie E.; Ngadi M. Comparative Evaluation of Thermogravimetric and Refractive Index Techniques in Determining Biodiesel Yield. J. Sustainable Bioenergy Syst. 2020, 10, 30–42. 10.4236/jsbs.2020.101003.

[ref25] Benedetti A.; Ilavsky J.; Segre C.; Strumendo M. Analysis of textural properties of CaO-based CO_2_ sorbents by ex situ USAXS. Chem. Eng. J. 2019, 355, 760–776. 10.1016/j.cej.2018.07.164.

[ref26] Margaretha Y. Y.; Prastyo H. S.; Ayucitra A.; Ismadji S. Calcium oxide from Pomacea sp. shell as a catalyst for biodiesel production. Int. J. Energy Environ. Eng. 2012, 3, 1–9. 10.1186/2251-6832-3-33.

[ref27] Liu X.; He H.; Wang Y.; Zhue S.; Piao X. Transesterification of soybean oil to biodiesel using CaO as a solid base catalyst. Fuel 2008, 87, 216–221. 10.1016/j.fuel.2007.04.013.

[ref28] Awogbemi O.; Inambao F.; Onuh E. I. Modification and characterization of chicken eggshell for possible catalytic applications. Heliyon 2020, 6, e05283 10.1016/j.heliyon.2020.e05283. 33102874 PMC7569345

[ref29] Naemchan K.; Meejoo S.; Onreabroy W.; Limsuwan P. Temperature Effect on Chicken Egg Shell Investigated by XRD, TGA and FTIR. Adv. Mater. Res. 2008, 55–57, 333–336.

[ref30] Tanabe K.; Hattori H.; Misono M.; Ono Y. New Solid Acids and Bases, 1st ed.; Elsevier: Amsterdam, 1990.

[ref31] Li Y.; Niu S.; Hao Y.; Zhou W.; Wang J.; Liu J. Role of oxygen vacancy on activity of Fe-doped SrTiO_3_ perovskite bifunctional catalysts for biodiesel production. Renewable Energy 2022, 199, 1258–1271. 10.1016/j.renene.2022.09.075.

[ref32] Calatayud M.; Ruppert A. M.; Weckhuysen B. M. Theoretical Study on the Role of Surface Basicity and Lewis Acidity on the Etherification of Glycerol over Alkaline Earth Metal Oxides. Chem.—Eur. J. 2009, 15, 10864–10870. 10.1002/chem.200900487. 19760708

[ref33] Hossain M.; Muntaha N.; Osman Goni L. K. M.; Jamal M. S.; Gafur M. A.; Islam D.; Fakhruddin A. N. M. Triglyceride conversion of waste frying oil up to 98.46% using low concentration K^+^/CaO catalysts derived from eggshells. ACS Omega 2021, 6, 35679–35691. 10.1021/acsomega.1c05582. 34984298 PMC8717581

[ref34] Li H. Y.; Wang Y.; Ma X.; Wu Z.; cui P.; Lu W.; Liu F.; Chu H.; Wang Y. A novel magnetic CaO-based catalyst synthesis and characterization: enhancing the catalytic activity and stability of CaO for biodiesel production. Chem. Eng. J. 2020, 391, 123549 10.1016/j.cej.2019.123549.

[ref35] Dhankhar S.; Bhalerao G.; Ganesamoorthy S.; Baskar K.; Singh S. Growth and comparison of single crystals and polycrystalline brownmillerite Ca_2_Fe_2_O_5_. J. Cryst. Growth 2017, 468, 311–315. 10.1016/j.jcrysgro.2016.09.051.

[ref36] Zhang N. H.; Xue H.; Hu R. The activity and stability of CeO_2_@CaO catalysts for the production of biodiesel. RSC Adv. 2018, 8, 32922–32929. 10.1039/C8RA06884D. 35547696 PMC9086313

[ref37] Guo W.; Sun W.; Lv L.-P.; Kong S.; Wang Y. Microwave–assisted morphology evolution of Fe–based metal–organic frameworks and their derived Fe_2_O_3_ nanostructures for Li–ion storage. ACS Nano 2017, 11, 4198–4205. 10.1021/acsnano.7b01152. 28334522

[ref38] Liu J.; Liu M.; Chen S.; Wang B.; Chen J.; Yang D. P.; Zhang S.; Du W. Conversion of Au(III)-polluted Waste Eggshell into Functional CaO/Au nanocatalyst for Biodiesel Production. Green Energy Environ. 2022, 7, 352–359. 10.1016/j.gee.2020.07.019.

[ref39] Loryuenyong V.; Rohing S.; Singhanam P.; Kamkang H.; Buasri A. Artificial Neural Network and Response Surface Methodology for Predicting and Maximizing Biodiesel Production from Waste Oil with KI/CaO/Al_2_O_3_ Catalyst in a Fixed Bed Reactor. ChemPlusChem 2024, 89 (9), e202400117 10.1002/cplu.202400117. 38771717

[ref40] Kilic S.; Toprak G.; Ozdemir E. Stability of CaCO_3_ in Ca(OH)_2_ solution. Int. J. Miner. Process. 2016, 147, 1–9. 10.1016/j.minpro.2015.12.006.

[ref41] Wu H.; Zhang J.; Wei Q.; Zheng J.; Zhang J. Transesterification of soybean oil to biodiesel using zeolite supported CaO as strong base catalysts. Fuel Process. Technol. 2013, 109, 13–18. 10.1016/j.fuproc.2012.09.032.

[ref42] Afsharizadeh M.; Mohsennia M. Catalytic synthesis of biodiesel from waste cooking oil and corn oil over zirconia-based metal oxide nanocatalysts. React. Kinet., Mech. Catal. 2019, 128, 443–459. 10.1007/s11144-019-01622-9.

[ref43] Daramola M. O.; Mtshali K.; Senokoane L.; Fayemiwo O. M. Influence of operating variables on the transesterification of waste cooking oil to biodiesel over sodium silicate catalyst: A statistical approach. J. Taibah Univ. Sci. 2016, 10, 675–684. 10.1016/j.jtusci.2015.07.008.

[ref44] Hsiao M.-C.; Lin C.-C.; Chang Y.-H. Microwave irradiation-assisted transesterification of soybean oil to biodiesel catalyzed by nanopowder calcium oxide. Fuel 2011, 90, 1963–1967. 10.1016/j.fuel.2011.01.004.

[ref45] Prokaewa A.; Smith S. M.; Luengnaruemitchai A.; Kandiah M.; Boonyuen S. Biodiesel production from waste cooking oil using a new heterogeneous catalyst SrO doped CaO nanoparticles. J. Met., Mater. Miner. 2022, 32, 79–85. 10.55713/jmmm.v32i1.1149.

[ref46] Anastopoulos G.; Dodos G. S.; Kalligeros S.; Zannikos F. CaO loaded with Sr(NO_3_)_2_ as a heterogeneous catalyst for biodiesel production from cottonseed oil and waste frying oil. Biomass Convers. Biorefin. 2013, 3, 169–177. 10.1007/s13399-012-0070-2.

[ref47] Degirmenbasi N.; Coskun S.; Boz N.; Kalyon D. M. Biodiesel synthesis from canola oil via heterogeneous catalysis using functionalized CaO nanoparticles. Fuel 2015, 153, 620–627. 10.1016/j.fuel.2015.03.018.

[ref48] Rabelo S. N.; Oliveira L. S.; França A. S. Biodiesel production from microwave irradiated reactor using homogeneous and heterogeneous catalysis. Engenharia Térmica 2018, 17, 18 10.5380/reterm.v17i1.62254.

[ref49] Helwani Z.; Zahrina I.; Amraini S. Z.; Sianturi R. I.; Idroes G. M.; Muslem; Idroes R. CaO from chicken eggshell supported on activated carbon and KOH (CaO/C/KOH) as catalyst for biodiesel production from off grade palm oil. IOP Conf. Ser.:Mater. Sci. Eng. 2021, 1087, 012053 10.1088/1757-899X/1087/1/012053.

[ref50] Khatibi M.; Khorasheh F.; Larimi A. Biodiesel production via transesterification of canola oil in the presence of Na–K doped CaO derived from calcined eggshell. Renewable Energy 2021, 163, 1626–1636. 10.1016/j.renene.2020.10.039.

[ref51] Camacho-Valencia F.; Vázquez-Rodríguez G.; Tavizón-Pozos J. A. Catalytic evaluation of eggshell-based calcium methoxide over Al_2_O_3_ for biodiesel generation from waste cooking oil. Biofuels, Bioprod. Biorefin. 2024, 18, 1873 10.1002/bbb.2684.

[ref52] Buasri A.; Kamsuwan J.; Dokput J.; Buakaeo P.; Horthong P.; Loryuenyong V. Green synthesis of metal oxides (CaO-K_2_O) catalyst using golden apple snail shell and cultivated banana peel for production of biofuel from non-edible *Jatropha Curcas* oil (JCO) via a central composite design (CCD). J. Saudi Chem. Soc. 2024, 28, 101836 10.1016/j.jscs.2024.101836.

[ref53] Palitsakun S.; Koonkuer K.; Topool B.; Seubsai A.; Sudsakorn K. Transesterification of Jatropha oil to biodiesel using SrO catalysts modified with CaO from waste eggshell. Catal. Commun. 2021, 149, 106233 10.1016/j.catcom.2020.106233.

