# Supplementary material for: Biodiesel Production Using K–Sr/CaO and CaO Catalysts Derived from Eggshells by Canola Oil Transesterification
Source: ACS Omega. 2025 Feb 16;10(7):6827–38. doi: 10.1021/acsomega.4c09118 (PMC11866205; doi:10.1021/acsomega.4c09118)
Supplement: Supplementary file 1 — ao4c09118_si_001.pdf [file ao4c09118_si_001.pdf]

* Supplementary Material

Supporting Information

Biodiesel production using K-Sr/CaO and CaO catalysts derived from eggshells by canola oil transesterification

Jesús Andrés Tavizón-Pozos [1], Humberto Cervantes-Cuevas [2], Germán Gustavo Garcia-Camacho [2], Gerardo Chavez-Esquivel [2]*, and Dwight Roberto Acosta-Najarro [3]

[1] *Investigadores por México SECIHTI-Área Académica de Química, Departamento de Ciencias Básicas, Universidad Autónoma Metropolitana Azcapotzalco, Av. San Pablo No. 420, Nueva el Rosario, Azcapotzalco, Ciudad de México, 02128, México.*

[2] *Área de Química, Departamento de Ciencias Básicas, Universidad Autónoma Metropolitana Azcapotzalco, Av. San Pablo No. 420, Nueva el Rosario, Azcapotzalco, Ciudad de México, 02128, México.*

[3] *Instituto de Física, Universidad Nacional Autónoma de México, Circuito de la Investigación Científica, Ciudad Universitaria, Coyoacán, Ciudad de México, 04510 México.*

Biodiesel was produced in a batch reactor using a 50 mL flat-bottom flask placed within a heat jacket, equipped with magnetic stirring and connected to a water-cooled reflux system, which included a condenser, as illustrated in Figure S1.

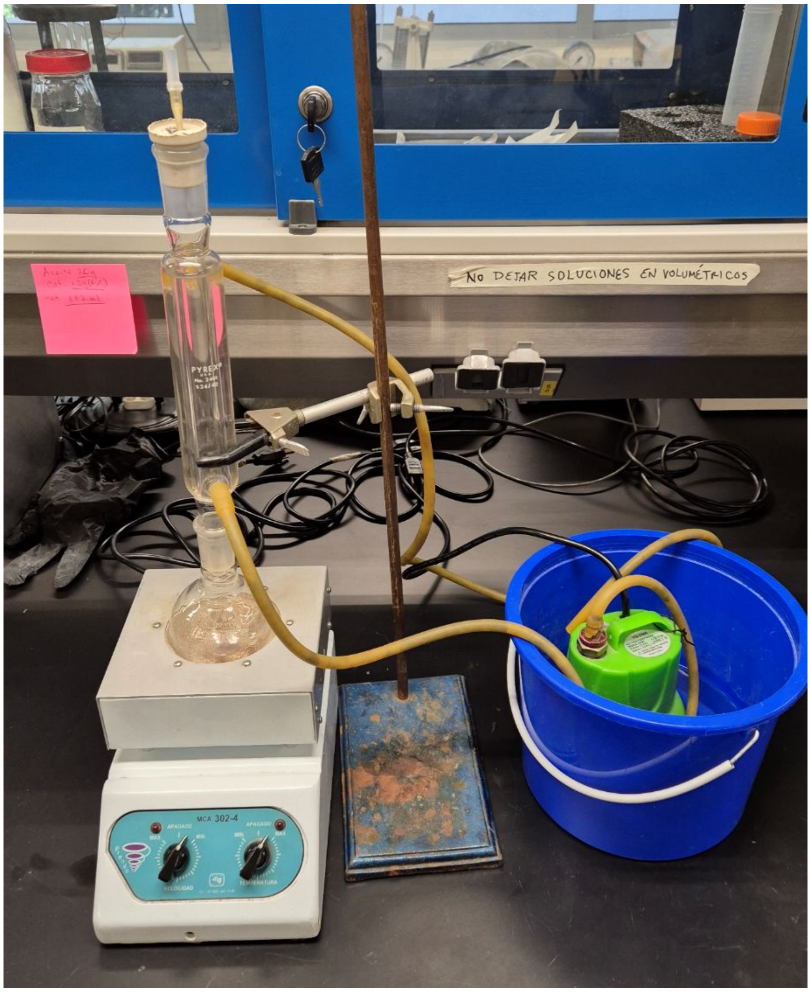

Figure S1. Picture of the reactor used for transesterification reactions.

The biodiesel density was determined a standardized pycnometer with 10 mL of sample at 20 °C. At 40 °C, viscosity was measured using an Ostwald viscometer (K=0.0025 mm^2/s^2). The saponification number (SN, mg_{KOH}/g) was determined by mixing 1.5 g of biodiesel with 20 mL of a 0.25 N KOH solution in ethanol and heating it in a water bath. It was then titrated with a 0.1 N HCl aqueous solution using phenolphthalein as an indicator, with water as a reference. The acid volume used was recorded, and the calculation was performed according to Equation 1:

$$SN = \frac{([KOH]\cdot V_{KOH}\cdot [HCl]\cdot V_{HCl})\cdot (56.1\ g\ eq^{-1})}{m_{sam}} \quad (1)$$

where [KOH] and [HCl] are the base and acid concentration (eq/L), respectively, V_{KOH} and V_{HCl} are the spent volumes, and m_{sam} is the sample weight.

For the acid number (AN, mg_{KOH}/g), 5.5 g of biodiesel was dissolved in 20 mL ethanol and titrated with a 0.1 N KOH solution using phenolphthalein as an indicator. The AN was calculated following Equation 2:

$$AN = \frac{[KOH]\cdot V_{KOH}\cdot MW_{OA}\cdot 100}{m_{sam}}$$

(2)

where [KOH] is the KOH (eq/L) concentration, V_{KOH} is the KOH volume used, MW_{OA} is the oleci acid molecular weight (282.52 g/mol), and m_{sam} is the sample weight.

Figure S2 presents the XRD profile of the non-calcined recovered Sr-K/CaO catalyst. The diffraction pattern indicates the presence of the $CaCO_3$ (ICDD-024-0027) phase, with characteristic peaks observed at 23.13°, 29.40°, 36.04°, 39.40°, 43.24°, 47.32°, 48.56°, 57.48°, 60.69°, and 64.76°. Additionally, a distinct peak at 10.32°, corresponding to calcium methoxide ($Ca(OCH_3)_2$), aligns with its highest intensity as referenced in the ICDD-00-031-1574 card. (1)

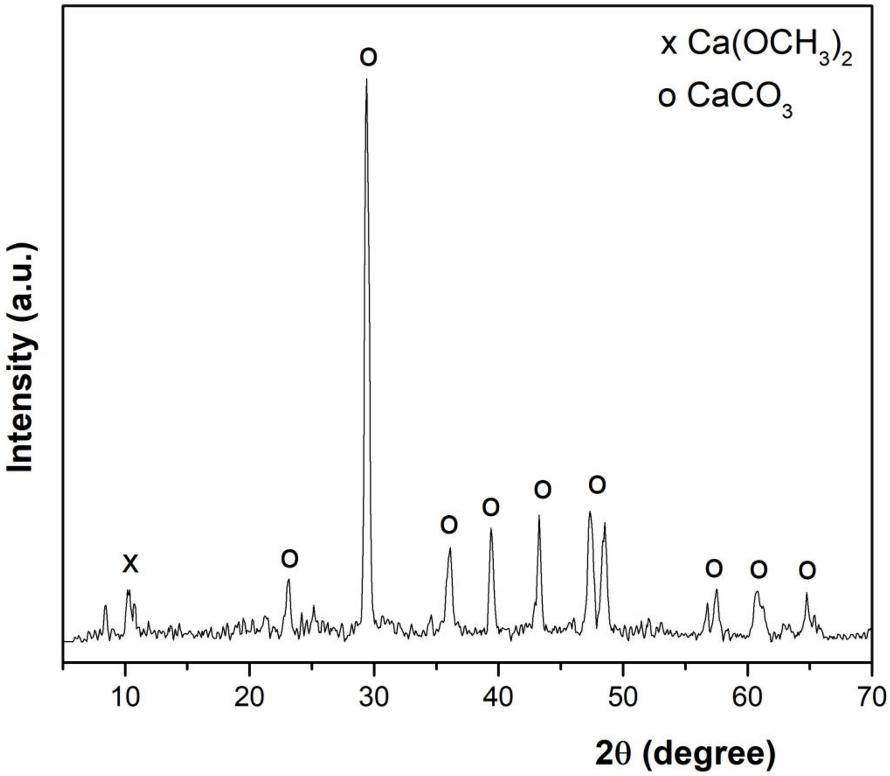

Figure S2. XRD pattern of the non-calcined recovered Sr-K/CaO catalyst after one cycle of reaction.

CaO and $Ca(OH)_2$ diffraction peaks were not detected, associated to a partial transformation to methoxide during the reaction, as seen in Figure 3. Additionally, the retention of the characteristic $CaCO_3$ peaks suggests that the catalyst remains stable after the transesterification reaction.

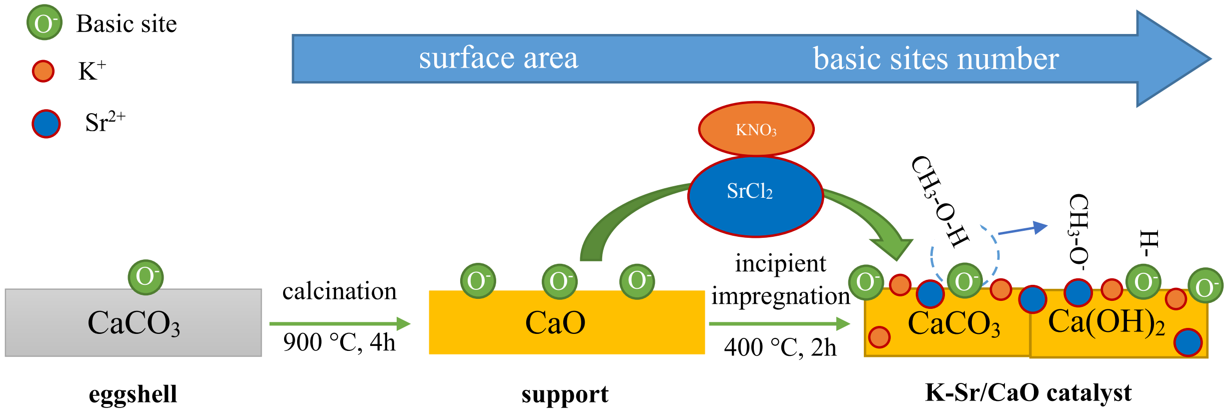

Figure S3. Schematic representation of the K-Sr/CaO catalyst crystalline phases and methoxyl group formation during the transesterification reaction for biodiesel production.

Figure S3 illustrates the predominant crystalline phases of the K-Sr/CaO catalyst. The transesterification reaction mechanism with methanol is also depicted, involving three key steps: *(i)* activation of the methanol molecule by O-, leading to the formation of the active methoxyl group (CH_3O^-); *(ii)* nucleophilic attack by CH_3O^- on the carbonyl group in the oil, followed by the formation of an intermediate, and *(iii)* the intermediate, containing a methoxyl group, is converted into a diglyceride, accompanied by the formation of fatty acid methyl ester. (2)

References

1. Chumuang, N.; Punsuvon, V. Response surface methodology for biodiesel production using calcium methoxide catalyst assisted with tetrahydrofuran as cosolvent. *Journal of Chemistry* **2017**, 2017, 4190818. DOI: 10.1155/2017/4190818

2. Zhang, H.; Li, H.; Xu, C. C.; Yang, S. Heterogeneously Chemo/Enzyme-Functionalized Porous Polymeric Catalysts of High-Performance for Efficient Biodiesel Production. *ACS Catalysis* **2019**, 9(12), 10990-11029. DOI:10.1021/acscatal.9b02748
